# Examining a Positive Variation of the Good Behavior Game with Head Start Children at Risk for Child Adversity and Externalizing Behavior Problems

**DOI:** 10.3390/children13050652

**Published:** 2026-05-06

**Authors:** Alexandra B. Gibson, Courtney P. D. Goldenberg, Malinda J. Colwell, Amelia E. Talley, Joaquín Borrego, Adam T. Schmidt

**Affiliations:** 1 Department of Pediatrics, University of Missouri-Kansas City School of Medicine, Children’s Mercy, Kansas City, MO 64108, USA; abgibson@cmh.edu; 2 Department of Psychological Sciences, Texas Tech University, Lubbock, TX 79409, USA; cgoldenb@ttu.edu (C.P.D.G.); amelia.talley@ttu.edu (A.E.T.); 3 Department of Human Development and Family Sciences, Texas Tech University, Lubbock, TX 79409, USA; malinda.colwell@ttu.edu; 4 School of Graduate Psychology, Pacific University, Forest Grove, OR 97116, USA; jborrego@pacificu.edu; 5 Center for Translational Neuroscience and Therapeutics, Texas Tech University Health Sciences Center, Lubbock, TX 79430, USA

**Keywords:** good behavior game, classroom management, head start, child adversity, early intervention, externalizing behaviors, preschool, young children

## Abstract

Background: Children participating in the Head Start program in the United States are predominantly from underserved groups, have increased rates of child adversity, and are at risk for externalizing behavior problems in the classroom. The Good Behavior Game (GBG) is an effective classroom management strategy for reducing disruptive and off-task classroom behaviors. However, previous research has not examined the GBG within the Head Start context. Methods: The current case–controlled study evaluated the effectiveness of the GBG in six Head Start children aged 3–5. Researchers conducted daily classroom behavioral observations of disruptive behaviors, and teachers completed pre- and post-treatment assessments of externalizing behaviors and social skills. Results: Study results showed the majority of target children exhibited reductions in at least one observed externalizing behavior and clinically significant improvements in teacher-reported externalizing behaviors, although most participants did not exhibit significant changes in social skills. Findings illustrate the effectiveness of structured classroom interventions, such as the GBG, for improving classroom behavioral compliance. Results have implications for teacher training and highlight the capacity for low-intensity interventions such as the GBG to have substantial impacts on classroom management within settings such as Head Start. Future research should endeavor to determine the optimal dosage and duration of the GBG, assess the effectiveness of teacher training to administer the GBG in their own classrooms, and evaluate if the current findings generalize to other contexts.

## 1. Introduction

Research associates adverse childhood experiences (ACEs) such as child maltreatment and household dysfunction with multiple deleterious outcomes, including higher rates of mental health disorders, academic difficulties, and increased involvement in the juvenile and adult justice systems [1,2,3]. Moreover, the impact of ACEs can reach across generations with parental ACEs exerting powerful downstream effects on offspring, especially during the preschool period [2,4,5]. A significant consequence of early ACEs exposure is increased rates of externalizing psychopathology, such as oppositional defiant disorder, conduct disorder, and attention deficit hyperactivity disorder. These risks may emerge as early as the preschool period [2,3] and may be particularly salient for preschoolers from underserved populations, such as children participating in the Head Start program in the United States.

Externalizing behaviors, such as aggression, noncompliance, hyperactivity, impulsivity, and destructive behaviors, occur frequently in preschool-aged children and are more common among underserved populations such as children participating in Head Start [6]. Preschoolers who exhibit externalizing behaviors at school are also at increased risk for experiencing relational difficulties with their peers and teachers, poorer academic outcomes, and are more likely to experience other childhood adversities, including child maltreatment and household dysfunction [2,3,7,8,9]. Children with co-occurring academic and behavior problems at entry into first grade have been shown to be more likely to (a) need special education services, (b) exhibit poorer performance on math and reading tests, (c) utilize mental health services, and (d) fail to graduate high school [10].

Research identifies externalizing behaviors as the most problematic type of classroom behavior for preschool teachers to manage. Head Start teachers, in particular, report a need for additional training to effectively manage externalizing behaviors in their classrooms [11]. One type of highly effective classroom behavior management strategy for children of all ages is group classroom contingencies [12]. Interdependent group contingencies apply the same contingency to all group members, with consequences applied based on the collective behavior of the group [13]. Research shows that these methods result in lower levels of disruptive behaviors than other types of group contingencies [14]. They are particularly effective given the equal distribution of responsibility among group members to perform at the expected level [12,15]. Single case experimental designs demonstrate interdependent group contingencies result in reductions in externalizing behaviors exhibited by preschoolers in Head Start classrooms [16,17] and other preschool settings [18,19]. Yet, interdependent group contingencies remain relatively understudied for preschool-aged children, especially those in underserved settings such as Head Start [12,20].

### 1.1. Good Behavior Game

One well-supported interdependent group contingency strategy is the Good Behavior Game (GBG) [21] and its positive variation (the GBG-R). The GBG-R (R indicates the use of positive reinforcement) emphasizes delivering reinforcement to teams of students engaging in positive behaviors while placing disruptive behaviors on extinction (e.g., not delivering attention or points when these behaviors occur). Evidence indicates the GBG-R is effective with children in a variety of grade levels, including preschool [19] and kindergarten [22,23,24,25]. Moreover, although Swiezy and colleagues’ 19] study suggests that GBG-R can increase compliance rates of preschool dyads, other research on preschool-aged children is scant. Additional studies are needed to clearly demonstrate the effectiveness of the GBG-R to reduce disruptive behaviors, such as off-task, inappropriate, or aggressive behaviors in preschoolers, particularly children in low-resource classrooms such as Head Start [16,17,22,24,25,26,27,28,29]. Finally, many previous studies using the GBG-R base their conclusions on the effectiveness of the intervention based upon visual inspection of observational data rather than statistical approaches designed for small-N research, such as time series analysis. Limitations of visual inspection include higher Type I error rates, particularly in the context of serial dependent data [30], and unreliable results across raters [31]. Thus, evaluating GBG-R using a time series analysis approach would greatly strengthen the evidence regarding the utility of the GBG-R for reducing externalizing behaviors in preschoolers.

### 1.2. Current Study

Given the limited resources available within the Head Start setting, the significant difficulties reported by Head Start teachers in managing externalizing behaviors, and the significant impact that externalizing behavior problems have on academic success, there is a critical need to identify interventions that are scalable, measurable, reproducible, and effective. Research suggests that the GBG-R may be a viable, relatively easily implemented intervention that could be used in the Head Start setting, although the existing literature is lacking in demonstrating its effectiveness in this setting or with such a young population. The current study evaluated the effectiveness of GBG-R using a single-case time-series multiple baseline research design [32]. Specifically, we hypothesized that GBG-R using randomized rewards would reduce classroom externalizing behaviors and increase classroom compliant behaviors across six preschool children enrolled in Head Start who exhibited elevated externalizing behavior problems. We measured behavior change using a combination of both direct behavioral observation and psychometrically valid teacher assessments of externalizing and prosocial behaviors.

## 2. Method

### 2.1. Participants

#### 2.1.1. Participant Recruitment

Families and teachers were recruited to participate in research as a part of a larger study taking place at a Head Start preschool in a medium-sized city in West Texas.

#### 2.1.2. Family Recruitment

Families were recruited at the local Head Start center for approximately 4 weeks by trained undergraduate and graduate research assistants. Caregivers who consented to participate were assigned a participant identification number (PID) and then given a packet containing measures and a demographic form marked with the corresponding PID.

#### 2.1.3. Teacher Recruitment

Teachers were recruited to participate during an after-school meeting during which the researcher described the role of teachers in the study and provided an opportunity for teachers to ask questions. Interested teachers completed a consent form and were given packets containing measures for each child in their classroom whose parents had consented and returned the pretreatment research packet.

**Identification of Target Children.** Scores on the Externalizing Problems composite of the Behavior Assessment System for Children—3rd Edition Teacher (BASC-3 TRS-P) were used to identify six target children with elevated levels of externalizing behaviors. Specifically, only children with Externalizing Problems T-scores ≥ 60 with validity indices in the acceptable range were included in this study. Exclusionary criteria included children with parent-reported diagnoses of intellectual or developmental disability or autism spectrum disorder. Additionally, no more than three target children were selected from any one classroom, and no pairs of siblings were selected.

Upon review of the screening data, 10 (20%) of the 50 participating children were eligible to participate in the current study with a T-score ≥ 60 T on the Externalizing Problems scale of the BASC-3 TRS-P. One of the 10 children was ineligible to participate due to a parent-reported diagnosis of autism spectrum disorder. Of the nine remaining children, five were in Classroom One, two were in Classroom Two, one was in Classroom Three, and one was in Classroom Four (please see Appendix A for a more detailed description of each participating classroom). Two of the five children in Classroom One had high Externalizing Problems T-scores on the BASC-3 TRS-P with validity indices in the *Extreme Caution* range, suggesting that the teacher may have over-reported these children’s externalizing behaviors. Thus, the next three children in Classroom One with elevated Externalizing T-scores were chosen as target children. Overall, six children (66% male) aged 3–5 were identified as target children for this study. Additionally, six control children, matched by age and sex and whose Externalizing Problems T-scores fell within the “normal” range (i.e., T-score of 41–59), were identified to blind the teacher rater to the identity of the target child. No data beyond teacher report measures were obtained for these control participants to reduce teacher response burden. Please see Table 1 for a summary of participant demographic data and Table 2 for a summary of parent- and teacher-reported BASC-3 Externalizing Problems scores.

Caregivers and teachers were incentivized to participate with cash compensation. Caregivers were compensated $20 for completing the pre-treatment measures and $20 for post-treatment screening measures, regardless of how many questions were answered. Caregivers who did not return the packet and, thus, voluntarily withdrew were not compensated. Teachers were compensated $5 per measure that they completed. Of note, the current study is limited to the analysis of teacher and observer ratings of behavior occurring within the classroom and during the intervention, respectively. Research indicates that teacher and clinician ratings have good convergence with each other and parent reports but may also yield important discriminant information [33,34,35]. As a result, they are important to use in combination to yield the most comprehensive view of child behavior. Finally, teacher ratings employed in the current investigation have been used in other small sample studies and found to accurately capture the behaviors of individual children in the classroom setting [36,37].

## 3. Measures

### 3.1. Behavior Assessment System for Children, Third Edition, Teacher Rating Scales, Preschool

Teachers’ perceptions of children’s adaptive and problem behaviors were assessed with the Behavior Assessment System for Children, Third Edition, Teacher Rating Scales, Preschool (BASC-3 TRS-P) at pre- and post-treatment [38]. The BASC-3 TRS-P is a broadband assessment of emotional and behavioral problems in young children aged 2–5, which has been normed with a national, representative sample of children. It is composed of 105 items, and teachers rate the extent to which an item reflects the child within the last several months on a 4-point Likert scale ranging from Never, Sometimes, Often, to Almost Always. Scores on the Externalizing Problems composite scale were the main outcome variable assessed with the BASC-3 TRS-3.

### 3.2. Revised Edition of the School Observation Coding System

The Revised Edition of the School Observation Coding System (REDSOCS) [39,40] is a direct observation system designed for measuring child disruptive behaviors in school settings that has been validated with preschool (*M* age = 4.8 years) children. The REDSOCS utilizes interval coding to measure the frequency of common externalizing behaviors during structured classroom activities. Relevant REDSOCS codes for this study included *inappropriate* and *off-task* behavior codes [39]. Undergraduate and graduate research assistants were trained to conduct REDSOCS observations using a combination of didactic instruction regarding the observational coding methods and category definitions, videotape coding, and in vivo coding of preschool children until they met at least 80% agreement when compared to a reliable coder, which is standard for REDSOCS coding reliability [40,41,42,43]. Interrater reliability was calculated after each live coding session using the REDSOCS Reliability Form. All coders demonstrated adequate reliability during live coding prior to the start of data collection (*M* percent agreement = 97.0%).

### 3.3. REDSOCS Observational Procedures

Each REDSOCS observation occurred daily during large-group activities. During the baseline phase, REDSOCS observations were conducted for each of the target children during the entirety of large group time, approximately 10–15 min. Each behavior category was observed and coded using interval coding methods. In classrooms with multiple target children (i.e., Classrooms One and Two), observations were conducted by multiple coders to capture the individual behavior of all target children. Coders observed different children each day according to a predetermined, randomized schedule to reduce observer drift and to limit observer bias by minimizing the likelihood that individual observers would develop affinities towards specific children and thus rate their behavior as more on task or less disruptive than others. During the intervention phase, REDSOCS observations began at the start of the game and continued for a minimum of 10 min or until the game ended.

### 3.4. Inter-Rater Reliability

The researchers randomly collected inter-rater reliability (IRR) data with a second coder for 58 (50.4%) of the 113 completed REDSOCS observations. IRR was calculated as the percent agreement for occurrences per category using the REDSOCS Reliability Form. Across all seven codes, *M* agreement was 92.00% (range = 79.72–100%). Percent agreement for each individual coding category is reported in Table 3.

### 3.5. Sutter-Eyberg Student Behavior Inventory-Revised

The Sutter-Eyberg Student Behavior Inventory–Revised (SESBI-R) [44] is a 38-item measure of teacher-reported child behavior problems occurring in a school setting that has been normed for children aged 2–16. Raw scores on the SESBI-R intensity scale were the main outcome variable assessed with the SESBI-R. The intensity scale measures the frequency with which externalizing behaviors occur and is rated on a seven-point Likert scale, 1 (Never) to 7 (Always). Scores range from 38 to 266, with higher scores associated with more problematic behaviors. Raw scores can be transformed into T-scores to aid comparison to normative data.

### 3.6. Social Skills Improvement System—Teacher Rating Scales

The Social Skills Improvement System—Teacher Rating Scales (SSIS-TRS) is an assessment of teacher-reported child social skills and problem behaviors that has been normed with a national sample of children aged 3–18 (Appendix A [45]). The SSIS-TRS scales relevant for preschoolers include Social Skills and Problem Behaviors. It is composed of 76 items relevant to preschool-aged children, and teachers rate the extent to which an item reflects the child within the last two months on a 4-point Likert scale ranging from Never, Seldom, Often, to Almost Always. Standard scores on the Social Skills scale were the main outcome variable assessed with the SSIS-TRS. The Social Skills scale is composed of Communication, Cooperation, Assertion, Responsibility, Empathy, Engagement, and Self-Control subscales. The Social Skills scale has demonstrated high internal consistency (*alpha* = 0.96–0.97), test–retest reliability (corrected *r* = 0.82), and moderate inter-rater reliability (corrected *r* = 0.68) with children aged 3–18 [45], and good convergent and discriminant validity [45].

## 4. Procedures

### 4.1. Design

A single-subject multiple-baseline across individuals design was utilized for this study. This type of design involves beginning baseline data collection at the same time, establishing a stable trend in observed behaviors, and staggering the start of the intervention phase across classrooms [32,46]. The number of baseline observations did not exceed 15 days due to limitations related to the number of data points per phase for simulation modeling analysis [47,48,49].

### 4.2. Baseline

At baseline, lead teachers in each of the three classrooms were asked to complete the SESBI-R on each target child and their matched controls in their classroom to assess baseline levels of child externalizing behaviors. Matched controls were selected by age and gender to mask the identity of the target child from the teacher. During this phase, teachers were instructed to conduct their large-group classroom activities and use their preferred behavior management strategies as they usually would, while coders conducted daily REDSOCS observations on each target child.

**Preparation for GBG-R.** During baseline, the researcher met with each set of teachers to discuss the intervention and explained that teachers should continue their normal classroom lessons while ignoring any rule violations during the GBG-R. The researcher collaboratively worked with teachers to choose reinforcers garnered from other GBG-R studies (i.e., stickers, scented lip balms, bubbles). The researcher also encouraged teachers to reduce access to the identified reinforcers outside of the intervention phase to increase the potency of the reinforcers.

At this time, the researcher also collaborated with the teachers to identify the GBG-R rules and to divide each class into teams. Each rule was represented on a poster board using a combination of words and pictographs. Please see Table 4 for a summary of each classroom’s rules and rewards. Three GBG-R teams (Red, Blue, Green) were identified for each classroom by considering the classroom seating chart and equally distributing target children into separate teams. This resulted in teams of five or six children across all three classrooms.

### 4.3. Intervention

The intervention methods used in this study were based on the Caught Being Good Game (CBGG) as described by Wright & McCurdy [25] and Wahl and colleagues [24]. The researcher led all GBG-R sessions and provided all the required materials needed to play the game, including a board with Velcro strips for each team and color-coded Velcro stars, and poster boards depicting each set of classroom rules. A researcher-led intervention was utilized due to the limited empirical support regarding the GBG-R as an evidence-based intervention for preschool children, as well as limitations related to time for completing teacher training. Each classroom started implementing the GBG-R at staggered time points in order to maintain the multiple baseline design. The GBG-R was played daily with the entire class during morning large-group classroom activities. REDSOCS observations were conducted during each GBG-R session. GBG-R sessions lasted approximately 10 min. This shorter game length is consistent with prior research implementing the GBG with preschoolers, who reported session lengths of 10–12 min [50].

**General GBG-R Procedures.** At the beginning of each (approximately) 10-min GBG-R session, the researcher announced the beginning of the game, identified each team, and discussed the posted rules of the game. The researcher then explained the contingency in place for each team to earn stars (i.e., all members of the team must be following the three rules for the team to earn a star) and the token criterion for winning each game session. The token criterion was also visually indicated on the game board by a gold piece of tape. While playing the game, the researcher was prompted to scan each team for rule-following behaviors using a vibrating timer application set on a variable-interval 3-min schedule. Each time the timer went off, the researcher scanned the room to identify which teams had all members displaying rule-following behaviors. Then, the researcher delivered a behavior-specific praise statement to rule-following teams and added a star to the board for those teams. Teams not engaging in rule-following behaviors were ignored and not given a star on the board. For example, if the researcher noticed a team all staying on their spots on the carpet, she might say, “Team Blue, great job staying on your spot on the carpet!” and then add a star to their spot on the board. This resulted in the researcher scanning the room and delivering praise and stars to rule-following teams approximately every 1 to 3 min.

The specific criterion (i.e., number of stars) to win the game was varied systematically across GBG-R sessions. The initial criterion was set at 2 stars per team in order to allow all teams to contact the contingency (i.e., winning the game). Once all three teams had won two consecutive games, the criterion was increased by 1 until it reached 4 stars total. Due to the 10-min time limit on GBG-R sessions, it was noted that it was not feasible for teams to consistently have the opportunity to earn more than 4 stars per game.

At the end of the game, the researcher announced the end of the game, reviewed the number of stars earned by each team, and announced which teams won the game that day. The researcher randomly selected a card from the reward box to determine the reinforcer for that session and then delivered the selected reinforcer to each team of children who won the game. Teams that did not win the game were reminded that they would have another chance to work with their team to obey the game rules and try to win the game the next day. Anecdotally, the majority of children in each classroom seemed motivated to earn the rewards offered during GBG-R sessions. This was evidenced by children expressing excitement about different stickers offered and encouraging their peers to smell the scent of the lip balm they chose. Periodically, a child expressed disinterest in a reward. These children were reminded they could win a different reward during the next session, and they were encouraged to try to win the game with their team again next time.

Classroom One completed 14/15 scheduled GBG-R sessions, with one session canceled due to an incident that required the children to be evacuated from the classroom for cleaning. Classroom Two completed 14/15 GBG-R sessions, with one session canceled due to a previously scheduled movie day. Classroom Three completed 13/14 GBG sessions, with one session canceled due to a previously scheduled movie day. Meta-analytic data derived from SCED GBG research suggests that playing the GBG for more sessions is not associated with larger treatment effects, with many included studies reporting session numbers ranging from 14 to 20 sessions [51]. Thus, it was anticipated that a treatment effect would be identified despite the relative brevity of the intervention period.

**Implementation fidelity.** During the intervention phase, implementation fidelity of the researcher’s execution of the GBG-R was also assessed for 21.4% of GBG sessions in Classroom One, and 23.1% of GBG sessions in Classrooms Two and Three using an integrity check form. The integrity check form was completed by a graduate student observer who was trained in the GBG procedures while the researcher implemented the game. Integrity checks were completed during randomly selected sessions when a graduate student was scheduled to complete an observation (e.g., Tuesdays and Thursdays). Across all observed sessions, implementation fidelity was calculated as 98.7% in Classroom One and 100% in Classrooms Two and Three.

### 4.4. Data Analysis Methods

The current study used simulation modeling analysis of time series data to separately evaluate the effects of the GBG-R for each target child. Additionally, results of simulation modeling analysis were enriched by also providing data from standardized measures (reporting the reliable change index when possible) and reporting effect sizes.

### 4.5. Simulation Modeling Analysis

Simulation modeling analysis (SMA) is a statistical technique based on bootstrapping methods that can be used to analyze short data streams (i.e., 5–15 data points per phase) [47,48]. SMA phase effect analysis allows the researcher to investigate whether there is a meaningful change in the person’s symptoms from baseline to the intervention condition [47]. SMA also allows for the analysis of autocorrelated data, which is a common issue in clinical research and time-series data when tracking change over time for one person [47,48]. For this study, the AR estimate for the whole dataset was used to avoid an inflated Type I error rate [52]. In SMA, power is evaluated based on the number of data points and the AR of those data points, rather than the number of subjects [49]. With the total number of datapoints ranging from 22 to 26 and AR values ranging from −0.32 to 0.70, it appears that this study was adequately powered. Phase effect analysis was conducted using SMA Version 8.8.3 software [52].

**Missing data in SMA.** All six target children had some proportion of missing data throughout the course of the study. Teachers reported that several target students had attendance problems and often missed school due to illness or factors related to transportation availability, although specific reasons were not provided to the researchers. Missing data was handled using the Expectation-Maximization procedure (EM procedure) [53] using SPSS Statistical software Version 24.0 (IBM Corp., New York, NY, USA, 2016 [54]). This procedure is appropriate for missing data that is identified as missing completely at random (MCAR), with data streams with lag-1 estimates of AR falling below 0.80 and <40% missing data [55]. Preliminary research suggests that using maximum-likelihood procedures to replace missing data can result in fairly accurate estimates of phase-effect statistics [56]. In circumstances where the EM procedure was used to impute missing data, the SMA analyses were not conducted on the original dataset due to an insufficient number of data points to complete the SMA analysis.

**Percentage of nonoverlapping data.** The percentage of nonoverlapping data (PND) is a widely used statistic for effect size measurement in SCED research [57]. PND has been shown to be sensitive to treatment effects in short data streams and less affected by AR of the data points than other SCED effect size statistics [58]. PND was used to further evaluate the results for participants without sufficient data for SMA. An online calculator published by [59], which provides both the PND and a *p* value for PND for single-case A-B research data, was used. This calculator takes into account the number of data points in Phase A (i.e., baseline) and Phase B (i.e., intervention), and the number of data points higher (or lower) than the highest (or lowest) data point in the baseline phase. Thus, this calculator was used to evaluate the PND for each REDSOCS variable using the original dataset. PND is generally interpreted as follows: <50% = no observed effect, 50% to 70% = questionable effectiveness, 70% to 90% = effective, and >90% = very effective interventions [60].

**Reliable change index.** The reliable change index (RCI) is another method for evaluating whether changes in measured behavior as a result of an intervention are clinically significant [61,62]. This statistical method is viable when normative data for standardized assessments are available, which is the case for the SESBI-R [44] and the SSIS-TRS [45]. For example, this type of procedure is helpful in determining if the change in a child’s score on the measure has changed from a clinically significant level at pre-treatment (i.e., ≥60 T) to a subclinical level at post-treatment (i.e., <60 T). The RCI is generally considered clinically significant when the RCI value is greater than or equal to ±1.96 [61,62]. Please refer to Table 5 for a summary of RCI scores for this hypothesis.

## 5. Results

### 5.1. Summary of Overall Findings

Teachers completed the BASC-3 TRS-P, SESBI-R, and Social Skills scale of the SSIS-TRS for each target and matched-control child in their classroom at post-treatment to uphold participant masking. Please see Table 6 for a summary of GBG-R activities and seating arrangements by classroom. Two of the six matched control children had dropped out of the Head Start program, and, therefore, teachers did not complete post-treatment measures on these children. When examining demographic and pre-treatment measures of the control children, it was noted that of the two children who dropped out, their mothers had lower levels of education (i.e., high school diplomas), lower annual income (i.e., <$20,000), received several forms of social assistance (e.g., WIC, SNAP), and first became a parent during adolescence.

### 5.2. Inappropriate Behaviors

**Figure 1:**
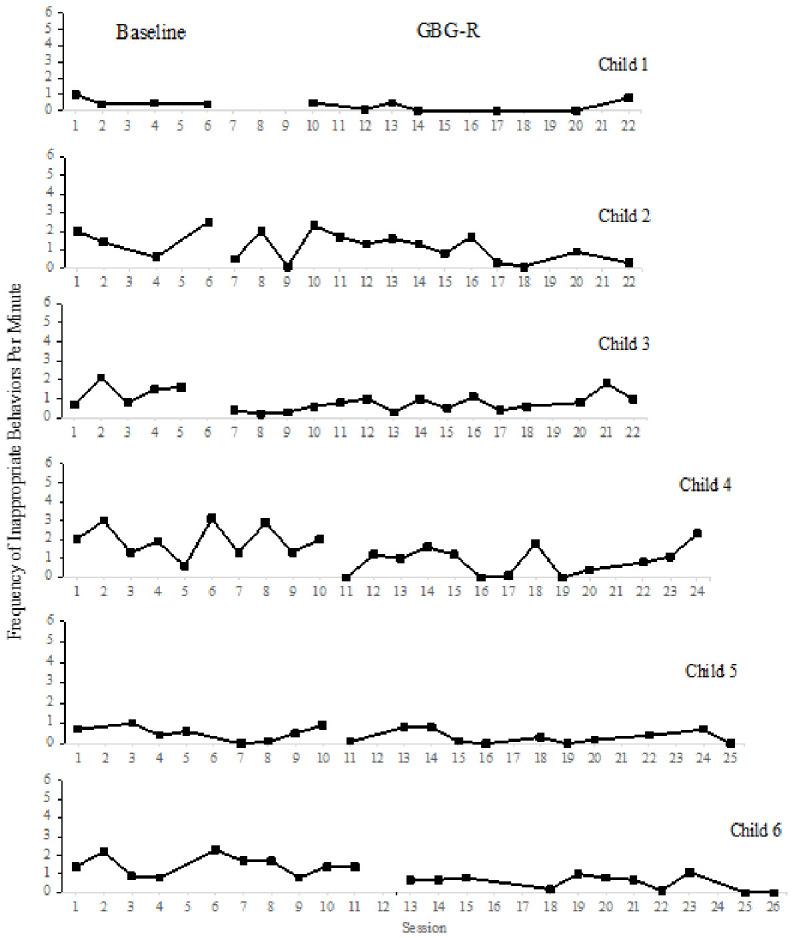
Decreases in Inappropriate Behaviors.

SMA was conducted to evaluate changes in inappropriate behaviors from baseline to treatment. Please see Table 7 and Table 8 for a summary of the results of SMA and PND. Please see Figure 1 for a visual summary of each participant’s REDSOCS Inappropriate code data. One participant’s data stream had too many missing data points to evaluate using SMA. Three (50.00%) students exhibited statistically significant reductions in inappropriate behaviors across the course of the intervention. Two (33.34%) students exhibited a PND that fell in the effective or very effective range.

### 5.3. Off-Task Behaviors

**Figure 2:**
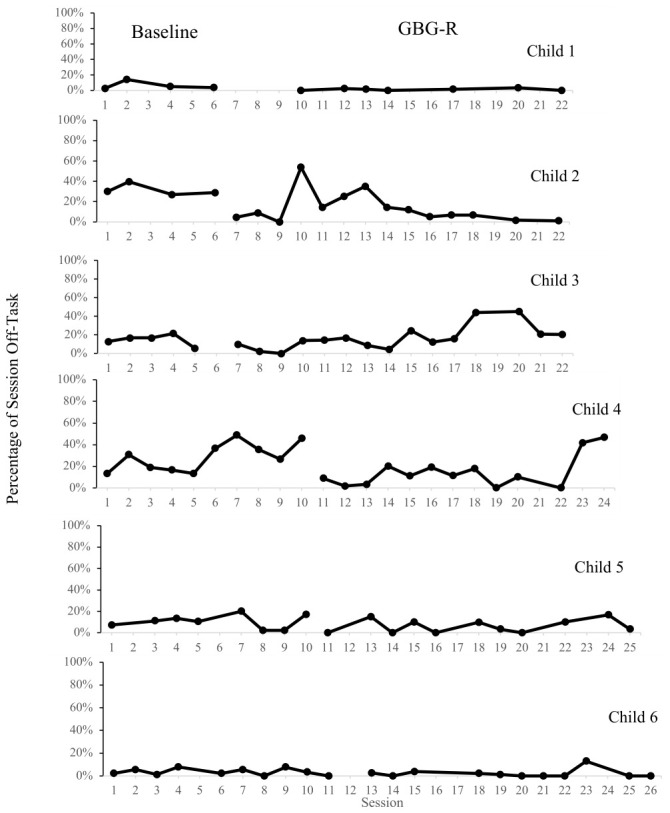
Decreases in Off-Task Behaviors.

SMA was conducted to evaluate changes in off-task behaviors from baseline to treatment. Please see Table 7 and Table 8 for summaries of the results of SMA and PND. Please see Figure 2 for a visual summary of each participant’s REDSOCS Off-Task code data. One participant’s data stream had too many missing data points to evaluate using SMA. Two (33.34%) students exhibited statistically significant reductions in off-task behaviors across the course of the intervention. Two (33.34%) students exhibited a PND that fell in the very effective range.

RCI values were calculated to evaluate change in teacher reports of child behavior problems from pre- to post-treatment as measured by the Intensity Scale of the SESBI-R and to examine change in social skills using the Social Skills scale of the SSIS-TRS. Please see Table 5 for a summary of RCI scores. Across the six target children, five students’ SESBI-R *Intensity* scores fell within the clinically significant range (>131) at pretreatment, suggesting these children were exhibiting elevated levels of externalizing behaviors prior to the start of the intervention. Overall, four (66.67%) target children exhibited clinically significant reductions in teacher-reported SESBI-R scores, from baseline to treatment, as measured by the RCI. However, when examining posttreatment scores, four children were rated as having clinically significant SESBI-R Intensity scores at posttreatment. This suggests that while the GBG-R had a clinically significant impact on externalizing behaviors, these children were continuing to exhibit high overall levels of problem behaviors. RCI analyses of Social Skills generally did not indicate any significant changes in social skills between pre- and post-treatment, except for one child who exhibited clinically significant improvement in social skills. This indicates that the GBG-R may not substantially impact a child’s social skills, at least during the time frame of the current investigation. Please see Appendix A for detailed information regarding participating classrooms and the results for individual child participants.

## 6. Discussion

Overall, the results of this study lend support to findings of previous research examining interdependent group contingencies to reduce preschool children’s disruptive behaviors in Head Start settings [16,17]. This study also extends the current literature on the utility of using GBG-R procedures with preschoolers [19] by demonstrating that it is a viable intervention for reducing inappropriate and off-task behaviors in classrooms of preschool children, in addition to preschool dyads.

Results of SMA indicated a variable pattern of change in externalizing behaviors in response to the GBG-R across target children. However, each of the five (83.33%) target children with sufficient data for SMA exhibited statistically significant reductions in either observed inappropriate or off-task behaviors from baseline to treatment. These data are especially salient given the lack of effect size data reported in GBG-R literature. This is likely attributable to the use of SCED research rather than large N designs. To date, effect sizes have only been reported in two meta-analyses [51,63].

Clinically significant reductions in teacher-reported externalizing behaviors were also found in this study. Four (66.67%) target children exhibited clinically significant reductions in teacher-reported SESBI-R scores from baseline to treatment as measured by the RCI, further supporting the positive impact of the GBG-R on externalizing behaviors. Further, the impact of the GBG-R in terms of general reductions in behavioral problems has not been presented in the previous literature, as data collection is conducted primarily during intervention sessions in the form of direct observation. These preliminary results indicate that the GBG-R resulted in generalized improvements in externalizing behaviors in most participants. These findings extend the present literature base by using rigorous statistical procedures appropriate for small-n research (SMA and PND) and psychometrically valid assessments of behavior (BASC-TRS), demonstrating that the GBG-R may be a viable, low-cost method for addressing behavioral concerns within the Head Start classroom and other low-resource settings.

Externalizing behaviors were still noted by teachers in several target children. When closely evaluating the data for the two participants who did not exhibit clinically significant reductions in SESBI-R scores (i.e., Child Two and Child Six), it was noted that both had very elevated SESBI-R and BASC-3 TRS-P scores at pre-treatment in comparison to other target children. In addition, these children had increased parental involvement at school and teacher-reported referrals for their levels of emotional and/or behavioral dysregulation during the course of the study. It is possible that teacher bias on post-treatment rating scales (i.e., SESBI-R, SSIS-TRS) may have contributed to the mixed results for these participants, particularly due to the lack of a matched control subject for Child Six at post-treatment. However, it is also possible that other events (e.g., stress within the home setting, peer challenges, etc.) may have influenced the degree to which these children were engaging in externalizing behaviors throughout the course of the study. Nonetheless, these results may also imply that the GBG-R may be an insufficient group intervention approach for children with substantially higher levels of clinically significant externalizing behaviors (e.g., those at risk for diagnosis of an emotional or behavioral disorder). Instead, the GBG-R may be better conceptualized as a universal prevention strategy to address mild-to-moderate behavioral concerns in the preschool setting as opposed to an intervention for children with more severe externalizing behavior concerns. Further investigation regarding potential moderators of treatment response, such as the presence of neurodevelopmental disorders (e.g., attention-deficit/hyperactivity disorder) or a history of traumatic stress, would be beneficial.

### 6.1. Research Implications

The current study is the first study with which we are aware to examine the effectiveness of the GBG-R within a Head Start (i.e., low-resource, preschool classroom setting) while also employing a more rigorous methodological approach to data analysis. As such, the present investigation contributes valuable evidence underscoring the effectiveness of the GBG-R for improving disruptive behaviors in preschoolers participating in the Head Start program. The application of a longitudinal, single-subject, multiple-baseline design in this study allowed for the investigation of the impact of the GBG-R on disruptive behaviors for children with clinically significant levels of behavior problems at pre-treatment. Moreover, this study is the first investigation of this type to use SMA and to explicitly report effect size data using PND. These methods provide important context regarding the magnitude of the effect of the GBG-R, which was in the range of “effective” or “very effective” in the current study. Lastly, the use of psychometrically valid and reliable assessment procedures contributes novel information to the present literature base regarding the impact of the GBG-R on target children’s overall levels of externalizing behaviors, whereas previous GBG-R studies have presented visual inspection of observational data as the sole outcome measure. Therefore, future studies should continue to utilize rigorous statistical approaches designed specifically for small-n studies in combination with psychometrically valid and reliable assessment procedures to best measure treatment outcomes.

### 6.2. Clinical Implications

The results of this study expand upon prior work from traditional (i.e., response cost) GBG research with preschoolers [50,64] by supporting the use of interdependent group contingencies with preschoolers without the need for significant procedural modifications used in previous research [19].. Additionally, these results extend findings from other reports that an interdependent group contingency, like the GBG-R, can be successfully used with preschool-aged children, including those from underserved/low-resource settings, such as the children participating in Head Start, to reduce the occurrence of externalizing behaviors [16,17]. In fact, there was some evidence that the GBG-R results may have generalized to other times of the school day, given the reductions in overall SESBI-R scores for several target children. Novel results such as these have not been reported in other GBG-R studies, likely due to the lack of the collection of psychometrically sound teacher-report data at pre- and post-treatment.

Limited improvements in teacher ratings of child social skills (i.e., cooperation, assertion, responsibility, empathy, and self-control) from pre- to post-treatment were noted across five out of six target children. This pattern of results may be explained by the absence of direct teaching, prompting, and positive reinforcement for the prosocial behaviors captured by this measure. Additionally, the limited duration of the intervention may have been insufficient to result in the acquisition of social skills. Future studies aiming to directly improve social skills may benefit from identifying target behaviors specifically related to prosocial behaviors.

Despite the lack of effect on social skills observed in the present investigation, our findings are particularly salient given the low-resource, high-risk preschool sample included in this study. Children enrolled in Head Start services are more likely to be exposed to a variety of risk factors associated with behavioral problems [65], to exhibit higher rates of externalizing behaviors than other groups of preschoolers [66,67], and their teachers are more likely to be inadequately prepared to effectively manage their behavior problems [11]. This study highlights an excellent opportunity for providing low-cost, straightforward tools that Head Start teachers can use to effectively manage disruptive behaviors in their classrooms and suggests these strategies may improve overall behavioral adjustment–at least for children with mild to moderate externalizing behaviors.

### 6.3. Limitations and Future Directions

Although we currently have encouraging preliminary data supporting the GBG-R, the nature of this empirical approach limits the generalizability of these results. The small sample size of six participants and predominantly male sample may also have influenced the prevalence of externalizing behaviors. However, male preschoolers are at high risk for engaging in off-task and disruptive behaviors [3]; thus, the male predominance of our sample may simply reflect the population at greatest risk rather than being a limitation in the study sample. Therefore, future studies should replicate these findings using larger samples of preschoolers, include a more balanced study sample of male and female children, and use a randomized control trial (RCT) design. Further, these results are also limited in generalizability given the researcher-led intervention approach. Presently, no GBG or GBG-R studies have been conducted with preschool teachers, themselves, acting as game facilitators [19,50,64]; however, numerous GBG-R studies have successfully used teacher implementors with older populations (e.g., [22,23,26,27,28,68]), making this a logical next step. Examining the impact of teacher-led GBG-R sessions on preschoolers’ disruptive behaviors, in addition to collecting teacher and student social validity data, would also increase the external validity of the GBG-R (i.e., by examining the feasibility and effectiveness of an intervention delivered directly by teachers in settings with limited resources).

Additionally, several issues arose related to blinding during data collection. Although it was not feasible to blind REDSOCS coders to the study phase, this was at least partially accounted for by randomly assigning coders to each target child for both baseline and GBG-R data collection sections. Despite blinding teachers to the identity of target children in their classroom via the use of matched controls, two matched control children dropped out of the Head Start program before the end of data collection. Thus, it is possible that these teachers’ post-treatment responses were biased (e.g., teachers’ perceptions of target children or their expectations for the intervention). Future studies would benefit from blinding teachers and investigators to the identity of target children when possible (e.g., comparing the GBG-R to an active control).

Future studies should also consider examining the impact of the GBG-R on children with disruptive classroom behavior but subclinical levels of externalizing behaviors, to determine if this intervention can effectively help regulate this relatively large group of children. This would provide important information regarding the possibility of using the GBG-R as a prevention-intervention for children who are not yet exhibiting substantial externalizing behaviors but may still be at higher risk for future problems. Moreover, inclusion of children with specific comorbidities or environmental risks known to exacerbate externalizing problems (e.g., attention problems, neurocognitive deficits, callous-unemotional traits, or certain environmental risks) would also be beneficial in determining the generalizability of the current findings to other groups of high-risk preschool children [37,69,70]. It would also be informative to examine the impact of the game over longer periods of time, such as an academic semester or year. Examining the effectiveness of the GBG-R over a longer time scale would also provide clarity regarding the sustainability/durability of any treatment effects and of using the GBG-R as a long-term, teacher-implemented behavior management strategy.

## 7. Conclusions

The current study examined the impact of the GBG-R on the level of externalizing behaviors in Head Start preschoolers based on classroom observations and teacher-report data. Results indicated that the GBG-R demonstrated effectiveness in reducing at least some disruptive behaviors. Interestingly, findings also suggested that participating children may demonstrate improvements in their overall levels of externalizing behaviors outside of the context of the GBG-R intervention. Children with moderate levels of behavioral difficulties appeared to benefit the most from the intervention, although children with more severe behavioral problems continued to exhibit clinically significant problems following the intervention.

The methodological innovations used in the present investigation (e.g., use of quantitative statistical procedures and psychometrically valid instruments) drastically increase the scientific rigor, improve generalizability, and facilitate replicability of these findings for future GBG-R studies. Additionally, this study provides preliminary evidence of the potential effectiveness of using a low-cost behavior management strategy to reduce levels of externalizing behaviors in at-risk preschoolers. If replicated in other venues and if feasibility is demonstrated for teacher-delivered interventions, the GBG-R has the potential to improve classroom management and disruptive behavior among preschool-aged children, especially those in low-resource settings such as Head Start. Moreover, if durable, these improvements may also hold promise for increasing academic readiness and improving long-term academic, behavioral, and social outcomes for this vulnerable group of children.

## Figures and Tables

**Table 1:** Participant Demographic Data.

Child	Age	Sex	Race	Classroom
1	3	Female	Hispanic	1
2	3	Male	Black/Hispanic	1
3	3	Male	White	1
4	5	Male	Black	2
5	5	Female	Hispanic	2
6	4	Male	Black/Hispanic	3

**Table 2:** Participant Parent- and Teacher-Reported BASC-3 Externalizing Problems Scores.

	BASC-3 TRS-P EXT	
Child	Pre	Post
1	78 **	62 *
2	75 **	70 *
3	63 *	56
4	71 **	69 *
5	64 *	59
6	71 **	87 **

Note. BASC-3 TRS-P = Behavior Assessment System for Children, Third Edition, Teacher Rating Scales, Preschool. BASC-3 PRS-P = Behavior Assessment System for Children, Third Edition, Parent Rating Scales, Preschool. EXT = Externalizing Problems composite. * Denotes T-score falls in the “At Risk” range (i.e., >60 T). ** Denotes T-score falls in the “Clinically Significant” range (i.e., >70 T).

**Table 3:** Inter-rater Reliability of REDSOCS Observations.

REDSOCS Code	*M* Percent Agreement	Range
Appropriate	84.16	59–100
Inappropriate	84.24	59–100
Compliance	95.34	78–100
Noncompliance	98.84	91–100
No Compliable Command Given	94.33	72–100
On-Task	89.79	68–100
Off-Task	91.40	71–100
Not Applicable	97.86	80–100

Note. REDSOCS = Revised Edition of the School Observation Coding System.

**Table 4:** Classroom Rules and Rewards for GBG-R.

Classroom	GBG-R Rules	Rewards
1	Stay on spot on carpet Keep hands to yourself Listen to the teacher	Stickers Bubbles Scented lip balms
2	Stay at seat at table Keep hands to yourself Share materials with teammates	Stickers Pencils Bubbles Scented lip balms
3	Stay on spot on carpet Keep hands to yourself Use an inside voice	Stickers Bubbles Scented lip balm

Note. GBG-R = Good Behavior Game-Reinforcement.

**Table 5:** Summary of Reliable Change Index (RCI) Scores.

	SESBI-R Intensity		
Child	Pre	Post	RCI
1	172	133	−5.01 *
2	188	195	0.90
3	181	132	−6.30 *
4	170	76	−12.09 *
5	105	62	−5.53 *
6	210	242	4.11

Note. SESBI-R = Sutter-Eyberg School Behavior Inventory—Revised. * Denotes clinically significant change in desired direction (i.e., decrease in SESBI-R Intensity score).

**Table 6:** Classroom Features.

	Age Range	*N*	GBG-R Activities	Seating Arrangements
Classroom 1	3–4	17	Reviewing their morning schedule, singing and dancing to music, and toileting time	Assigned spot on the carpet identified by a name tag and a picture of each child
Classroom 2	4–5	17	Writing practice, drawing, arts and crafts, and molding shapes with playdough	Assigned seat at their table, identified by a nametag and a photo
Classroom 3	3–4	16	Reviewing the calendar, alphabet, and vocabulary words; reading aloud; and dancing and singing to music	Assigned a colored square on the carpet

Note. *N* = number of children in the classroom. GBG-R = Good Behavior Game-Reinforcement.

**Table 7:** Descriptive Data and Results of Simulation Modeling Analysis of REDSOCS Off-Task Variable and Percent Nonoverlapping Data.

	Baseline				GBG-R						
Target Child	*N*	*n*	*M* (*SD*)	% Missing	*N*	*n*	*M* (*SD*)	% Missing	*AR*	Level Change	PND
1	6	4	0.07 (0.05)	33.33	16	7	0.01 (0.01)	56.25			93.75 ^^^^
2	6	4	0.32 (0.06)	33.33	16	14	0.14 (0.15)	12.50	0.21	−0.56 *	87.50 ^^^
3	6	5	0.15 (0.06)	16.67	16	15	0.17 (0.13)	6.25	0.19	0.08	18.75
4	10	10	0.29 (0.13)	0.00	15	13	0.15 (0.15)	13.33	0.33	−0.46	53.33
5	10	8	0.10 (0.06)	20.00	15	11	0.06 (0.06)	26.67	−0.26	−0.35 *	26.67
6	12	10	0.04 (0.03)	16.67	14	11	0.02 (0.04)	21.43	−0.20	−0.25	0.00

Note. REDSOCS = Revised Edition of the School Observation Coding System. GBG-R = Good Behavior Game–Reinforcement. *N* = total number of days in each phase; *n* = number of completed observations per phase; *AR* = Lag-1 autocorrelation, calculated for the entire data stream after replacing missing data using the expectation-maximization procedure. Level change results are presented as correlation coefficients (Pearson’s *r*). PND = Percent Nonoverlapping Data. * *p* < 0.05. ^^^ = PND fell in *effective* range, ^^^^ = PND fell in *very effective* range.

**Table 8:** Descriptive Data and Results of Simulation Modeling Analysis of REDSOCS Inappropriate Variable and Percent Nonoverlapping Data.

	Baseline				GBG-R						
Target Child	*N*	*n*	*M* (*SD*)	% Missing	*N*	*n*	*M* (*SD*)	% Missing	*AR*	Level Change	PND
1	6	4	0.58 (0.29)	33.33	16	7	0.27 (0.32)	56.25			81.25 ^^^
2	6	4	1.63 (0.82)	33.33	16	14	1.06 (0.74)	12.50	−0.16	−0.36	31.25
3	6	5	1.34 (0.59)	16.67	16	15	0.72 (0.15)	6.25	0.18	−0.55 *	56.25
4	10	10	1.94 (0.53)	0.00	15	13	0.88 (0.75)	13.33	0.02	−0.57 **	33.33
5	10	8	0.53 (.035)	20.00	15	11	0.31 (0.32)	26.67	0.12	−0.36	0.00
6	12	10	1.46 (0.53)	16.67	14	11	0.55 (0.40)	21.43	0.52	−0.75 **	71.43 ^^^

Note. REDSOCS = Revised Edition of the School Observation Coding System. GBG-R = Good Behavior Game–Reinforcement. *N* = total number of days in each phase; *n* = number of completed observations per phase; *AR* = Lag-1 autocorrelation, calculated for the entire data stream after replacing missing data using the expectation-maximization procedure. Level change results are presented as correlation coefficients (Pearson’s *r*). PND = Percent Nonoverlapping Data. * *p* < 0.05. ** *p* < 0.01. ^^^ = PND fell in *effective* range.

## Data Availability

Data are not available for public access.

## Supplementary Materials

Details on classroom and individual participant results can be found in the supplementary materials, including supplementary tables with descriptive data for each REDSOCS variable and the results of SMA. The following supporting information can be downloaded at: https://www.mdpi.com/article/10.3390/children13050652/s1, Table S1: Descriptive Data and Results of Simulation Modeling Analysis of REDSOCS Variables for Child 4; Table S2: Descriptive Data and Results of Simulation Modeling Analysis of REDSOCS Variables for Child 5; Table S3: Descriptive Data and Results of Simulation Modeling Analysis of REDSOCS Variables for Child 6.

## Appendix A. Classroom and Individual Participant Supplementary Information

### Appendix A.1. Description of Classrooms and Individual Participants

Classroom One was composed of 17 preschoolers aged 3–4 years. Three target children participated in the GBG in this classroom. GBG-R sessions took place during morning large group activities such as reviewing their morning schedule, singing and dancing to music, and toileting time. Each student had an assigned spot on the carpet, identified by a name tag and a picture of each child. The Red team was composed of five children, the Blue team was composed of six children, and the Green team was composed of six children. Partway through data collection, one child on the Blue team and one child on the Green team left the Head Start program, resulting in five children in each team.

### Appendix A.2. Child One

This 3-year-old Hispanic female’s data stream contained two (33.33%) missing data points during baseline and nine (56.25%) missing data points during the GBG-R for all REDSOCS variables. As the proportion of missingness for all variables exceeded 40% during GBG, none of the REDSOCS variables could be analyzed with SMA [25]. However, PND was calculated based on the original dataset (e.g., not including missing data imputed using the EM procedure) for each REDSOCS variable. Overall, based upon the results of PND, the GBG-R was effective at reducing this child’s externalizing behaviors and increasing her positive behaviors, but did not appear to provide a mechanism for the acquisition of more general social skills. Our inability to analyze this participant’s data using SMA is a limitation of this participant’s results and highlights the fact that these results should be interpreted with caution. Moreover, this participant had inconsistent engagement in the intervention, thereby obscuring whether other factors may have contributed to these findings (e.g., changes in behavioral management strategies at home).

### Appendix A.3. Child Two

A summary of this 3-year-old biracial male’s descriptive data for each REDSOCS variable and the results of SMA (including AR values) can be found in Table 7. It should be noted that mid-data collection, a teacher reported that this child had been referred for a psychiatric evaluation due to his levels of emotional and behavioral dysregulation at home and school. Thus, it is unclear if this participant received additional intervention services during the course of data collection, although this appears unlikely. This participant’s data stream contained two (33.33%) missing data points during baseline and two (12.50%) missing data points during treatment for appropriate, inappropriate, on-task, and off-task variables, and two (33.33%) missing data points during baseline and three (18.75%) missing data points during GBG-R for compliant and noncompliant variables. The variation in proportions of missingness across REDSOCS variables can be attributed to sessions in which teachers did not provide any commands. This level of missing data has been demonstrated to be acceptable for SMA when the EM procedure is utilized with datasets containing missing data proportions below 40% and AR values < 0.8 [25]. For this participant, AR values ranged from −0.19 to 0.22. Therefore, missing data was analyzed and replaced using the EM procedure. Little’s [71] MCAR test was nonsignificant for the entire data stream, χ^2^(4) = 0.23, *p* = 0.99, suggesting that missing data did not introduce bias into the analyses. PND was also calculated based on the original dataset (e.g., not including missing data imputed using the EM procedure) for each variable. Results indicate that the GBG-R was limited in effectiveness for reducing this child’s externalizing behaviors in the school setting, with the exception of his off-task behaviors. Similarly, although the GBG-R may have increased his ability to stay on-task during GBG-R sessions, it did not result in a general improvement in his social skills.

### Appendix A.4. Child Three

A summary of this 3-year-old Caucasian male’s descriptive data for each REDSOCS variable and the results of SMA is located in Table 8. This participant’s data stream contained one (16.67%) missing data point during baseline and one (6.25%) missing data point during treatment for appropriate, inappropriate, on-task, and off-task variables, and one (16.67%) missing data point during baseline and two (12.50%) missing data points during GBG-R for compliant and noncompliant variables. For this participant, AR values ranged from −0.32 to 0.19. Therefore, missing data was analyzed and replaced using the EM procedure. Little’s [71] MCAR test was significant for the entire data stream, χ^2^(4) = 17.32, *p* = 0.002, suggesting that missing data did introduce bias into the analyses. However, based on the teacher’s report, these missing variables can be attributed to two separate days of missed school due to illness. Thus, it was hypothesized that this data can still be considered to be missing at random and therefore testable using SMA. PND was also calculated based on the original dataset (e.g., not including missing data imputed using the EM procedure) for each variable. Overall, these results suggest that the GBG-R was effective for reducing this child’s inappropriate behaviors, in addition to his general level of externalizing behaviors per teacher report data. Similarly, the GBG-R seems to have led to improvements in both his general appropriate behaviors and his social skills in the classroom.

Classroom Two was composed of 17 preschoolers aged 4–5 years. Two target children participated in the GBG-R in this classroom. GBG-R sessions took place during large group activities such as writing practice, drawing, arts and crafts, and molding shapes with play-dough, seated at their assigned tables. Each student had an assigned seat at their table, identified by a nametag and a photo. The teachers had previously distributed children to three separate tables identified by three colors (e.g., red, blue, green) during classroom activities. As the students were already familiar with their table teams, the children were not redistributed across teams (target children were already equally distributed among tables). The Red team was composed of five children, the Blue team was composed of six children, and the Green team was composed of six children. No participants joined or left this classroom during the data collection period.

### Appendix A.5. Child Four

This 5-year-old Black male’s descriptive data for each REDSOCS variable and the results of SMA can be found in Supplemental Table S1. This participant’s data stream contained no missing data points during baseline and two (13.33%) missing data points during GBG-R for appropriate, inappropriate, on-task, and off-task variables, and two (20%) and eight (53.33%) missing data points for compliant and noncompliant variables during baseline and GBG-R, respectively. As the proportion of missingness for compliant and noncompliant behaviors exceeded 40%, these two variables could not be analyzed with SMA [25]. For the remaining variables, missing data was analyzed and replaced using the EM procedure, given AR values ranging from 0.02 to 0.33. Little’s [71] MCAR test was nonsignificant for the entire data stream, χ^2^(4) = 3.00, *p* = 0.56, suggesting that missing data did not introduce bias into the analyses. PND was also calculated based on the original dataset (e.g., not including missing data imputed using the EM procedure) for each variable. Overall, the GBG-R resulted in a reduction in this child’s inappropriate behaviors and his overall level of externalizing behaviors. However, while his appropriate behaviors did increase, his teacher-reported social skills worsened from baseline to treatment, suggesting limited effectiveness of the GBG-R for improving this child’s social skills.

### Appendix A.6. Child Five

This 5-year-old Hispanic female’s descriptive data for each REDSOCS variable and the results of SMA are located in Supplemental Table S2. This participant’s data stream contained two (20.00%) missing data points during baseline and four (26.67%) missing data points during treatment for appropriate, inappropriate, on-task, and off-task variables, and four (33.33%) missing data points during baseline and 13 (86.67%) missing data points during GBG-R for compliant and noncompliant variables. As the proportion of missingness for compliant and noncompliant variables exceeded 40% during GBG-R, these variables could not be analyzed with SMA [25]. However, missing data was analyzed and replaced using the EM procedure for the remaining four variables, and PND was calculated based on the original dataset (e.g., not including missing data imputed using the EM procedure) for each REDSOCS variable. For this participant, AR values ranged from −0.36 to 0.36. Little’s [71] MCAR test was nonsignificant for the entire data stream, χ^2^(4) = 0.38, *p* = 0.98, suggesting that missing data did not introduce bias into the analyses. Overall, these results indicate that the GBG-R resulted in reductions in this child’s off-task behaviors, in addition to her general level of externalizing behaviors while at school. However, only this child’s on-task behaviors increased from baseline to treatment, and her social skills decreased from baseline to treatment, indicating that the GBG-R was generally ineffective at increasing her prosocial behaviors or her general social skills.

Classroom Three was composed of 16 preschoolers aged 3–4 years. One target child participated in the GBG-R in this classroom. GBG-R sessions took place during morning large group activities on the carpet, including reviewing the calendar, alphabet, and vocabulary words; reading aloud; and dancing and singing to music. Each student had an assigned colored square on the carpet, but spots were not marked by names or pictures. The Red team was composed of five children, the Blue team was composed of six children, and the Green team was composed of five children. No participants joined or left this classroom during the data collection period.

### Appendix A.7. Child Six

This 4-year-old biracial male’s descriptive data for each REDSOCS variable and the results of SMA are located in Supplemental Table S3. Notably, throughout data collection, this child’s caregiver was repeatedly called to school due to his level of disruptive behavior throughout the day, and his caregiver requested a referral for psychological treatment. Based on information provided to the research team, it did not appear that he received significant behavioral therapy during the course of data collection. This participant’s data stream contained two (16.67%) missing data points during baseline and three (21.43%) missing data points during treatment for appropriate, inappropriate, on-task, and off-task variables, and two (16.67%) missing data points during baseline and four (28.57%) missing data points during GBG-R for compliant and noncompliant variables. This level of missing data has been demonstrated to be acceptable for SMA when the EM procedure is utilized with datasets containing missing data proportions below 40% and AR values <0.8 [25]. For this participant, AR values ranged from −0.75 to 0.26. Therefore, missing data was analyzed and replaced using the EM procedure. Little’s [71] MCAR test was nonsignificant for the entire data stream, χ^2^(4) = 0.72, *p* = 0.15, suggesting that missing data did not introduce bias into the analyses. PND was also calculated based on the original dataset (e.g., not including missing data imputed using the EM procedure) for each variable. These results suggest the GBG-R was ineffective at reducing this child’s overall level of externalizing behaviors, besides his level of inappropriate behaviors. Similarly, it does not appear that the GBG-R was an effective intervention for increasing his positive behaviors or general social skills, beyond an increase in his rate of appropriate behaviors. However, it should be noted that anecdotal observations of teacher-child interactions and information relayed to the researcher by the lead teacher suggest that this teacher had consistently negative perceptions of this child, which may have impacted her behavioral ratings. Relatedly, the matched control child for Child 6 was one of the two who dropped out, meaning this teacher was not blinded to the identity of this target child at post-treatment.
