# Supplementary material for: Examining a Positive Variation of the Good Behavior Game with Head Start Children at Risk for Child Adversity and Externalizing Behavior Problems
Source: Children (Basel). 2026 May 6;13(5):652. doi: 10.3390/children13050652 (PMC13204244; doi:10.3390/children13050652)
Supplement: Supplementary file 1 [file children-13-00652-s001.zip › children-4202947-supplementary.pdf]

Supplemental Table S1. Descriptive Data and Results of Simulation Modeling Analysis of REDSOCS Variables for Child 4.

	Baseline				GBG-R					
Variable	*N*	*n*	*M* (*SD*)	% Missing	*N*	*n*	*M* (*SD*)	% Missing	*AR*	Level Change
Inappropriate	10	10	1.94 (0.53)	0.00	15	13	0.88 (0.75)	13.33	.02	-.57**
Off-Task	10	10	0.29 (0.13)	0.00	15	13	0.15 (0.15)	13.33	.33	-.46
Appropriate	10	10	4.06 (0.84)	0.00	15	13	5.12 (0.75)	13.33	.02	.57**
On-Task	10	10	0.71 (0.13)	0.00	15	13	0.85 (0.15)	13.33	.33	.47

Note. REDSOCS = Revised Edition of the School Observation Coding System. GBG-R = Good Behavior Game – Reinforcement. *N* = total number of days in each phase; *n* = number of completed observations per phase; *AR* = Lag-1 autocorrelation, calculated for the entire data stream after replacing missing data using expectation-maximization procedure. Level change results are presented as correlation coefficients (Pearson's *r*). $^{*}p < .05$. $^{**}p < .01$. $^{***}p < .001$.

Supplemental Table S2. Descriptive Data and Results of Simulation Modeling Analysis of REDSOCS Variables for Child 5.

	Baseline				GBG-R					
Variable	*N*	*n*	*M* (*SD*)	% Missing	*N*	*n*	*M* (*SD*)	% Missin g	*AR*	Level Change
Inappropriate	10	8	0.53 (.035)	20.00	15	11	0.31 (0.32)	26.67	.12	-.36
Off-Task	10	8	0.10 (0.06)	20.00	15	11	0.06 (0.06)	26.67	-.26	-.35*
Appropriate	10	8	5.48 (0.35)	20.00	15	11	5.69 (0.32)	26.67	.12	.36
On-Task	10	8	0.90 (0.06)	20.00	15	11	0.94 (0.06)	26.67	-.26	.35*

Note. REDSOCS = Revised Edition of the School Observation Coding System. GBG-R = Good Behavior Game – Reinforcement. N = total number of days in each phase; n = number of completed observations per phase; *AR* = Lag-1 autocorrelation, calculated for the entire data stream after replacing missing data using expectation-maximization procedure. Level change results are presented as correlation coefficients (Pearson's *r*). $^{*}p < .05$. $^{**}p < .01$. $^{***}p < .001$.

Supplemental Table S3. Descriptive Data and Results of Simulation Modeling Analysis of REDSOCS Variables for Child 6.

	Baseline				GBG-R					
Variable	*N*	*n*	*M* (*SD*)	% Missing	*N*	*n*	*M* (*SD*)	% Missing	*AR*	Level Change
Inappropriate	12	10	1.46 (0.53)	16.67	14	11	0.55 (0.40)	21.43	.52	-.75**
Off-Task	12	10	0.04 (0.03)	16.67	14	11	0.02 (0.04)	21.43	-.20	-.25
Appropriate	12	10	4.54 (0.53)	16.67	14	11	5.45 (0.40)	21.43	.52	.75**
On-Task	12	10	0.96 (0.03)	16.67	14	11	0.98 (0.04)	21.43	-.20	.25

Note. REDSOCS = Revised Edition of the School Observation Coding System. GBG-R = Good Behavior Game – Reinforcement. N = total number of days in each phase; n = number of completed observations per phase; *AR* = Lag-1 autocorrelation, calculated for the entire data stream after replacing missing data using expectation-maximization procedure. Level change results are presented as correlation coefficients (Pearson's *r*). $^{*}p < .05$. $^{**}p < .01$. $^{***}p < .001$.
